# Muscle Quality Matters More Than Quantity in Hip Fracture Recovery

**DOI:** 10.1111/ggi.70388

**Published:** 2026-02-02

**Authors:** Po‐Chin Strong, Chao‐Chun Huang

**Affiliations:** ^1^ Department of Physical Medicine and Rehabilitation Far Eastern Memorial Hospital New Taipei City Taiwan; ^2^ Department of Physical Therapy and Assistive Technology National Yang Ming Chiao Tung University Taipei Taiwan

To the Editor,

Unno et al. [1] recently highlighted the importance of preoperative intramuscular adipose tissue content (IMAC) in early functional recovery after hip fracture.

In daily rehabilitation practice, we frequently encounter patients who demonstrate preserved systemic stability—adequate nutritional indices and stable vital parameters—yet remain unable to initiate effective gait despite intensive therapy. The findings by Unno et al. provide a compelling physiological explanation for this clinical discrepancy: a dissociation between “systemic metabolic reserve” (reflected by PhA) [2] and “locomotor mechanical integrity” (reflected by IMAC) [1].

The authors' observation that IMAC independently predicts walking recovery, while PhA does not, raises a critical question regarding our current therapeutic strategies: Are conventional resistance‐based rehabilitation paradigms sufficient for patients with high IMAC? [3] The presence of myosteatosis implies a qualitative deficit in motor unit recruitment and force transmission. Consequently, we propose that patients identified with high preoperative IMAC might require distinct interventions, such as targeted neuromuscular re‐education or high‐velocity power training, rather than standard strengthening protocols alone.

Finally, while the authors acknowledge the 2‐week follow‐up limitation, this early period is often just the beginning of the functional journey. In models like Taiwan's nationwide Post‐Acute Care (PAC) program, patients undergo high‐intensity rehabilitation for several weeks to months [4]. Whether IMAC retains its strong predictive value for ambulatory recovery during such prolonged, high‐intensity rehabilitation remains an important and clinically actionable question that warrants further investigation.

## Funding

The authors have nothing to report.

## Disclosure

The authors have nothing to report.

## Ethics Statement

The authors have nothing to report.

## Consent

The authors have nothing to report.

## Conflicts of Interest

The authors declare no conflicts of interest.

## Data Availability

Data sharing is not applicable to this article as no new data were created or analyzed in this study.
